# Polar Mapper: a computational tool for integrated visualization of protein interaction networks and mRNA expression data

**DOI:** 10.1098/rsif.2008.0407

**Published:** 2008-10-28

**Authors:** Joana P. Gonçalves, Mário Grãos, André X.C.N. Valente

**Affiliations:** 1 Unidade de Sistemas Biológicos, Biocant, 3060-197 Cantanhede, Portugal; 2 KDBIO Group, INESC-ID, 1000-029 Lisbon, Portugal; 3 IST, Technical University of Lisbon, 1169-047 Lisbon, Portugal; 4 Unidade de Biologia Celular, Biocant, 3060-197 Cantanhede, Portugal; 5 Centro de Neurociências e Biologia Celular, Universidade de Coimbra, 3004-517 Coimbra, Portugal

**Keywords:** protein interaction network, functional genomics, systems biology

## Abstract

Polar Mapper is a computational application for exposing the architecture of protein interaction networks. It facilitates the system-level analysis of mRNA expression data in the context of the underlying protein interaction network. Preliminary analysis of a human protein interaction network and comparison of the yeast oxidative stress and heat shock gene expression responses are addressed as case studies.

## 1. Overview

Progress in the reliability and throughput of protein physical interaction detection techniques (both experimental- (Rual *et al*. 2005; Stelzl *et al*. 2005; Gingras *et al*. 2007) and computational-wise (Valencia & Pazos 2002)) is gradually leading to the availability of more comprehensive, higher confidence protein interaction data (Bader *et al*. 2003; Xenarios *et al*. 2004; Mishra *et al*. 2006; Stark *et al*. 2006; Wu, X. *et al*. 2006). There is hope that such ‘interactome’ maps can serve as invaluable tools for biological research, in particular for more integrated system-level studies of biological processes and mechanisms (Uetz & Finley 2005). Notably, in this regard, a protein interaction network provides the natural context for interpreting large-scale gene expression data, as the latter can be viewed as the dynamical expression of different parts of the protein interaction network (Jansen *et al*. 2002; de Lichtenberg *et al*. 2005). Now, interactomes can be very large, with rough estimates placing the number of interactions in a human cell of the order of 200 000 (Hart *et al*. 2006). Therefore, for the potential benefits of interactome mapping projects to be realized, proper visualization of interaction data is essential (Hu *et al*. 2007). As an addition to currently available alternatives (Batagelj & Mrvar 1998; Enright & Ouzounis 2001; Breitkreutz *et al*. 2003; Shannon *et al*. 2003; Batada 2004; Hu *et al*. 2004; Lu *et al*. 2004; Hooper & Bork 2005; Iragne *et al*. 2005; Li & Kurata 2005; Meil *et al*. 2005; Baitaluk *et al*. 2006), we present a software application, Polar Mapper, designed for displaying protein interaction networks in a particularly informative fashion, termed a polar map (Valente & Cusick 2006). The software also allows gene expression data to be overlayed on the generated polar map for a visually integrated analysis of expression and interaction data. To exemplify the usefulness of Polar Mapper, we applied it to two case studies: (i) a preliminary analysis of the collection of human protein–protein interaction data obtained via high-throughput yeast two-hybrid (Y2H) assays by Rual *et al*. (2005), and (ii) a preliminary search for the relevant differences in the, *a priori* very similar (Gasch *et al*. 2000), expression responses of *Saccharomyces cerevisiae* to the distinct hydrogen peroxide and heat shock stresses.

## 2. Related tools

Several software applications are available for protein interaction network visualization and analysis (Batagelj & Mrvar 1998; Enright & Ouzounis 2001; Breitkreutz *et al*. 2003; Shannon *et al*. 2003; Batada 2004; Hu *et al*. 2004; Lu *et al*. 2004; Hooper & Bork 2005; Iragne *et al*. 2005; Li & Kurata 2005; Meil *et al*. 2005; Baitaluk *et al*. 2006). While typically representing proteins as nodes and interactions as edges, tools such as Pajek (Batagelj & Mrvar 1998), BioLayout (Enright & Ouzounis 2001), CNplot (Batada 2004), PINC (Lu *et al*. 2004) or Medusa (Hooper & Bork 2005) rely on distinct layout algorithms to produce alternative graphical representations of an interaction network. Spring-embedded, hierarchical, orthogonal, tree and circular are among the most widely used kinds of structures (Tollis *et al*. 1998). Layouts can be accomplished using a number of distinct approaches, ranging from simulated annealing or gradient-descent minimization of the energy of the representation (Enright & Ouzounis 2001; Li & Kurata 2005) to hierarchical clustering techniques that group the nodes according to their similarity (Lu *et al*. 2004). These techniques are commonly combined with heuristics, which are able to prune the often large set of output arrangements. Moreover, several additional criteria are considered when measuring the quality of the graphical positioning of graph elements: the number of crossing edges; the area occupied by the representation; or the distance between adjacent and non-adjacent nodes (Tollis *et al*. 1998). Osprey (Breitkreutz *et al*. 2003), Cytoscape (Shannon *et al*. 2003), VisANT (Hu *et al*. 2004), ProViz (Iragne *et al*. 2005) and BiologicalNetworks (Baitaluk *et al*. 2006) further enable the integration of biological data available at public repositories. Functional annotations can be loaded as node attributes from Gene Ontology (Ashburner *et al*. 2000) by Osprey, Cytoscape, VisANT and BiologicalNetworks. VisANT also supports GenBank (Benson *et al*. 2007) and SwissProt (Wu, C. *et al*. 2006) annotations. Edges can be complemented with information on pathways and interaction types provided by the KEGG (Ogata *et al*. 1999) database in both VisANT and BiologicalNetworks. The latter further accepts the interaction data from the BIND (Bader *et al*. 2003) and TransPath (Krull *et al*. 2006) databases. Moreover, VisANT enables the integration of homology information and the projection to orthologous genes, based on phylogenetic profiles available at COG (Tatusov *et al*. 2001). The superposition of gene expression data's additional node information is supported by both Cytoscape and BiologicalNetworks. Additional functionalities for querying, navigating and finding substructures in graphs using either clustering or common algorithms in graphs are also provided by these tools. Interaction network analysis tools are mostly available as standalone applications. BiologicalNetworks, VisANT and Osprey are the exceptions, the first being provided solely as a web-based server and the others supporting both standalone and online execution forms. While the majority of these software tools are implemented in Java, thus being compatible with a multitude of platforms, a number of them are restricted to either Windows (Batagelj & Mrvar 1998; Li & Kurata 2005) or UNIX environments (Enright & Ouzounis 2001).

## 3. Computational algorithms

Polar Mapper introduces an alternative graphical display of networks, designated a polar map, in an application that was developed to be a practical, useful auxiliary tool in biological research projects involving the analysis of protein interaction networks. Additional key features of the Polar Mapper software include: (i) a convenient method for navigating the network based on its modularity analysis, (ii) an optional visual superposition of gene expression data upon the interaction network display, (iii) the specification of the nodes' sizes as a way to encode further information in the visualization (for instance, the molecular weight of a protein, or the number of members in a protein complex, for cases in which nodes represent protein complexes, rather than individual proteins), (iv) the ability to save network information as text and export polar maps as raster (PNG) and vector (SVG and PDF) image files, and (v) the support for maintaining the data and manual annotations associated with a given network in a Polar Mapper session file, enabling users to conveniently have their network analysis work evolve along with their biological research project. Details on how to use the Polar Mapper software in practice are provided in the Polar Mapper guide, available at the Polar Mapper website (http://kdbio.inesc-id.pt/software/polarmapper).

We next provide a description of the polar map visualization algorithm (Valente & Cusick 2006) integrated in Polar Mapper. An overview of the key steps in the algorithm is shown in figure 1, while figure 2 shows an alternative, more detailed flow chart. Representing the proteins as nodes and the interactions between the proteins as links between those nodes, the question becomes where to place the nodes in the plane, in order to obtain as meaningful and visually clear representation as possible of the interaction network. Now, given that the position of each node has two degrees of freedom, this allows the encoding of two distinct types of information in the graph: one, we shall associate with the radial coordinates of the nodes; the other with their angular coordinates.

**Figure 1:**
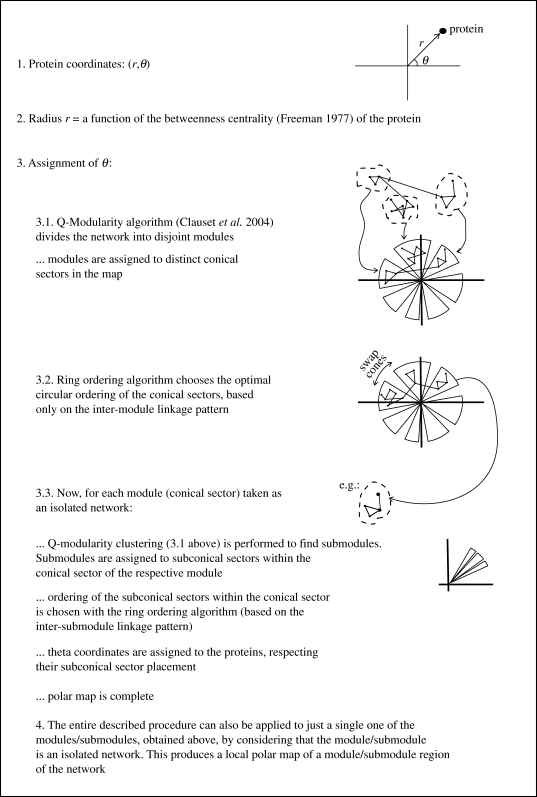
The polar map algorithm. Overview of the key steps in constructing a polar map display of a network.

**Figure 2:**
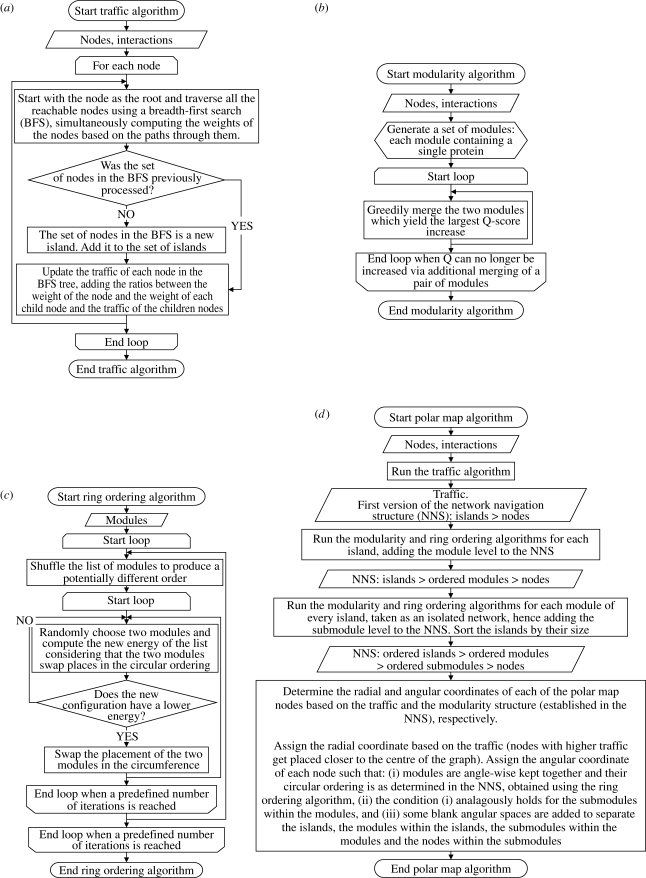
Detailed flow diagrams of the algorithms used to generate a polar map. (*a*) Traffic computes the betweenness centrality of each node (Freeman 1977; Newman & Girvan 2004) and divides disconnected groups of nodes in islands; (*b*) modularity generates the modules via the Q-modularity algorithm (Clauset *et al*. 2004) (*c*) The ring ordering procedure (Valente & Cusick 2006) finds a good ordering for the modules; and, finally, (*d*) the polar map algorithm calculates the polar coordinates of each node based on the results from (*a–c*).

The radial coordinate is used to introduce a mathematical hierarchical classification for the nodes based on their placement within the network (Brandes 2003). For this hierarchical classification, we choose the betweenness centrality measure (Freeman 1977). For a node, its betweenness centrality is defined as the total number of shortest paths (between any two other nodes in the network) that pass through it. In keeping with what is visually intuitive, we place higher betweenness centrality nodes closer to the centre of the graph and lower betweenness centrality nodes on the periphery of the graph. Owing to the long tail of the betweenness centrality value distribution (Goh *et al*. 2001), we use a logarithmic scaling, letting the radial coordinate of a node be proportional to log(max_BC_/node_BC_), where node_BC_ denotes the betweenness centrality of that node and max_BC_ the highest betweenness centrality in the network. Noting that the proteins range from those that function only within specific well-defined cellular processes to those that play more global, higher level functional roles, the inspiration for the above procedure lays in that, perhaps, this biological hierarchy in the role of proteins finds a correspondence in their placement within the mathematical, abstract protein interaction network. The true relevance and form of this parallel between the mathematical network betweenness centrality (or possibly an alternative centrality measure) of proteins and their ‘biological hierarchical centrality’ is still an open question (Coulomb *et al*. 2005; Hahn & Kern 2005; Joy *et al*. 2005; Estrada 2006; Junker *et al*. 2006). Regardless, at least, used in this fashion, it is very helpful in visually untangling large protein interaction graphs.

The angular placement of the nodes in the map is going to reflect the modular structure of the mathematical network, in the sense that it contains regions comparatively dense in links. The greedy algorithm of Clauset *et al*. (2004) is used to search for a partition of the network into disjoint modules that maximizes the modularity score
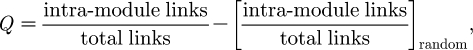

where the first term pertains to the network in question as it is, while the second assumes that the links in that network were randomized, subject to every node keeping its original degree. In other words, a high *Q*-score partitioning of the network guarantees that the number of within-module links is maximized with respect to a base random case, represented by the second term in the above formula. Note that the algorithm is not guaranteed to find the partition that yields the *Q* global maximum (Clauset *et al*. 2004). However, the so far significantly modular structure found in protein interaction networks (Spirin & Mirny 2003; Brun *et al*. 2004; Pereira-Leal *et al*. 2004; Nabieva *et al*. 2005; Valente & Cusick 2006; Wang & Zhang 2007) assures that, in practice, the partition found is probably not far off the optimal one. Combined with the fact that protein interaction networks are large and this algorithm's running time scales almost linearly in the number of nodes for sparse networks (such as protein interaction networks; Clauset *et al*. 2004), this makes it a good choice for the purpose at hand. This partitioning of the network is represented visually by allocating each module to a distinct angular region in the graph. That is, the angular coordinates of the nodes are assigned so that all the nodes in a given module fall within the same visual conical section. The biological importance of the mathematical partitioning of the network stems from evidence at present supporting that protein modules dense in physical interactions tend to correspond to biological functional modules in the cell (Spirin & Mirny 2003; Brun *et al*. 2004; Pereira-Leal *et al*. 2004; Nabieva *et al*. 2005; Valente & Cusick 2006; see Wang & Zhang (2007) for a different view).

Now, the above procedure still leaves the circular ordering of the modules in the graph undetermined. We would like to choose this ordering based solely on the linkage pattern across the modules, placing, to the extent that is possible, closer to each other modules that are in some sense more interconnected. Formally, this is done via the ring ordering algorithm (Valente & Cusick 2006), which works as follows. A function that associates an energy *E* with each potential circular ordering is defined. Given a circular ordering of the modules, let the distance between two modules be the shortest of the two possible distances between them around the circle (i.e. if they are next to each other, the distance is 1; if there is a module between them, the distance is 2, etc.). The energy for this circular ordering is then defined as
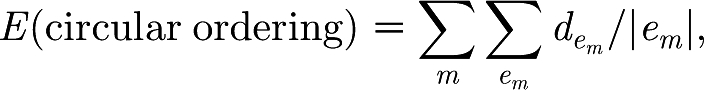

where *m* denotes a module in the network; *e*_*m*_ denotes an edge between module *m* and another module; 
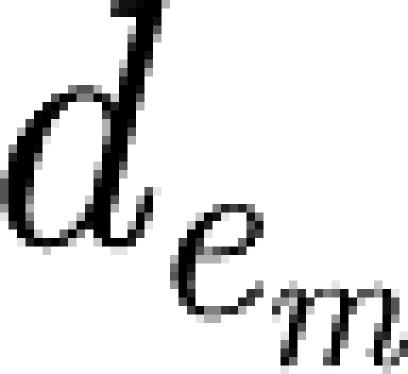
 denotes the distance between the modules connected by the *e*_*m*_ edge; and |*e*_*m*_| denotes the total number of edges between module *m* and other modules. The normalization by |*e*_*m*_| ensures that every module is given equal weight as far as determining the final arrangement. Now, the lower the energy of an ordering, the better the ordering is considered to be. The search for a low *E* ordering is done via a greedy procedure. Taking a random circular ordering as a seed, module position permutations are successively checked and performed if they yield a lower *E* circular ordering. The procedure is also repeated starting from different random seeds, with, eventually, the net lowest *E* circular ordering found being the chosen one. Note that the procedure does not guarantee that a global minimum for *E* is achieved. The angular ordering of the nodes within the angular section assigned to their module is further refined as follows: (i) the module is considered as an isolated network (by ignoring links from the module to the rest of the network), (ii) the *Q*-modularity-based partitioning is applied to the isolated module producing submodules, (iii) the ring ordering algorithm is applied to the linkage pattern between these submodules, producing an ordering of the submodules, (iv) the angular section of the module is divided among the submodules, respecting their ordering from (iii), and (v) within the angular section of a submodule, the angular ordering of its nodes is arbitrary. The motivation for this overall module ordering procedure is again that the density of connections between modules probably correlates with their biological functional closeness, which can be valuably reflected in the graphical display, at least to the extent allowed by the linear circular ordering constraint.

The above algorithm produces a polar map for an entire island (isolated graph) in the network (or for the entire network itself, simply by, as a pre-step, assigning separate angular sections to each island). However, it can be useful to visualize polar maps of specific regions in a network. A local module polar map is constructed in the same fashion, upon considering the given module as an isolated network. The modular breakdown into islands, modules and submodules has the additional advantage of providing a structured organization for navigating the network, and it is used for that purpose in the Polar Mapper software.

## 4. Biological case studies

### 4.1. Human interactome preliminary analysis

The Center for Cancer Systems Biology Human Interactome version 1 (CCSB-HI1) dataset (Rual *et al*. 2005) is one of the two first ever collections of human protein–protein interaction data experimentally obtained in a large-scale fashion (Rual *et al*. 2005; Stelzl *et al*. 2005). In that study, using the yeast two-hybrid assay in a high-throughput format, the products associated with approximately 8000 human genome open reading frames were systematically pairwise tested for possible physical interactions. This yielded approximately 2800 binary physical interactions. Note that the large-scale format of the assay is obtained at some cost; for instance, the assay is strictly binary (effects on the interaction of third-party proteins or post-translational modifications are not addressed) and so is its output (interaction detected/not detected, rather than a binding affinity type or other more complex characterization of the interaction). A basic question raised by the above extensive interactome mapping work is how to organize such a large raw dataset: how to grasp its overall structure and how to profitably turn the dataset into a useful aid in specific biological research problems. A more concrete fundamental question is whether indeed some form of functional organization of the cell is present at the level of the interactome, and, if it is, whether it is detectable in such a dataset, given the disputed reliability of the yeast two-hybrid technique (Hart *et al*. 2006) and the other assay limitations alluded to above.

As our first application of Polar Mapper, we use it as an auxiliary tool in a preliminary exploration of the CCSB-HI1 human interactome dataset. The reader is encouraged to load the associated session file of the human interactome, *HumanInteractome.pm*, and select ‘Island 1’ on Polar Mapper to follow along this analysis (see the file *session_instructions.pdf* in the electronic supplementary material for help). Henceforth, module and submodule IDs and names refer to the annotations in this session file. Figure 3 shows the largest connected component of the network. We analysed the generated modules in order to determine whether they reflected biological functions of the cell. Some modules are apparently not particularly functionally coherent, which may be explained by the fact that the data cover only a very small part of the interactome (of the order of 1% of the existing protein interactions are present in the dataset (Rual *et al*. 2005)). False-positive Y2H interactions may also account for some discrepancies (Rual *et al*. 2005). Nevertheless, several modules and submodules clearly show a theme, with the majority of their proteins possessing related functions or sharing common signalling pathways. We could identify the modules and/or submodules that are related to the regulation of transcription (mod. 17), housekeeping/biosynthetic pathways (submod. 31), cell proliferation/death and cancer (mod. 19), spliceosome/pre-mRNA splicing (submod. 92), cell division and cancer (submod. 104), cytoskeleton and protein scaffolding (submod. 52) and survival signalling (mod. 1).

**Figure 3:**
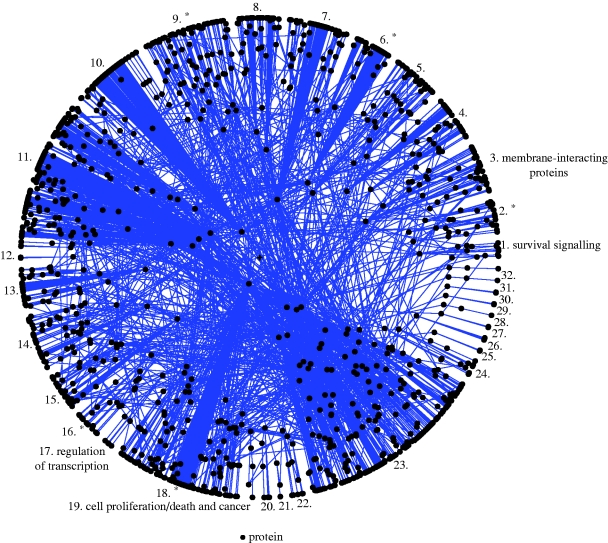
The human interactome. A polar map showing the largest connected component of the human interactome based on the Rual *et al*. (2005) yeast two-hybrid high-throughput protein–protein interaction dataset (1307 proteins and 2441 interactions). The modules are numbered from 1 to 32. Some of them were manually annotated (using Polar Mapper), reflecting the biological function of the proteins that constitute them. The modules marked with an asterisk contain annotated submodules.

We now describe an interesting module (mod. 3) we identified whose main theme is membrane-interacting proteins (figure 4). All its submodules contain membrane-interacting or transmembrane proteins. Three of the submodules should be highlighted in particular, as they are very coherent and can be further subcategorized as vesicular transport (I and II) and secretory pathway/membrane trafficking (figure 4). The vesicular transport I submodule contains well-known SNAREs (soluble *N*-ethylmaleimide-sensitive factor attachment protein receptors), known for their important role in diverse vesicle-mediated transport events: VAMP4 and VAMP3; syntaxin 4, 5 and 11; and SNAP23 and 25 (Hong 2005). NAPA, also known as α-SNAP, is involved in intra-Golgi transport (Hong 2005). SCGN was apparently an outsider, but a recent paper described that this protein binds directly to SNAP25 in response to calcium and may be involved in Ca^2+^-induced exocytotic processes (Rogstam *et al*. 2007). The SCGN–SNAP25 interaction was not present in the Rual *et al*. (2005) Y2H dataset used in this study. On the other hand, a SCGN–SNAP23 interaction was present. It remains to be seen whether this is an artefact of the Y2H screening due to the homology of the two SNAPs, or whether this interaction is biologically relevant. TAF6L is the only protein unrelated to vesicular transport present in this submodule. The second vesicular transport submodule we highlight contains the following: RABAC1, involved in vesicle formation from the Golgi complex and that interacts with SNARE complexes (Gougeon *et al*. 2002); RAB1A, involved in vesicular transport from ER to Golgi (Tisdale *et al*. 1992); RTN1, shown to bind to several SNAREs (Steiner *et al*. 2004); and SNX15, involved in endosomal trafficking (Barr *et al*. 2000; Phillips *et al*. 2001). It also contains DUSP12, which seems to be unrelated to this submodule's general theme: it is the human orthologue of the *S. cerevisiae* YVH1 protein tyrosine phosphatase (Muda *et al*. 1999) and is thought to negatively regulate the members of the mitogen-activated protein (MAP) kinase superfamily. The remaining two proteins of the submodule have unknown functions. The third submodule we highlight is composed of proteins involved in secretory pathway/membrane trafficking. Reticulons (RTN1–4; RTN3 and RTN4 are contained in this submodule) are associated with the endoplasmic reticulum and are involved in either neuroendocrine secretion or membrane trafficking in neuroendocrine cells (reviewed in Oertle & Schwab 2003). All members of this family have been shown to interact with and modulate BACE1 (a protease involved in the secretory pathway and a therapeutic target in Alzheimer's disease). Furthermore, the overexpression of any reticulon protein significantly reduces the production of amyloid-beta (He *et al*. 2004). RTN3 was described to be involved in membrane trafficking and protein transport between the ER and Golgi (Wakana *et al*. 2005). RAB33A is a small GTPase Rab family GTP-binding protein that localizes to dense-core vesicles and may be involved in vesicle transport during exocytosis (Tsuboi & Fukuda 2006). LRCH4 is a poorly characterized leucine-rich protein that contains a carboxyl terminus that may act as a membrane anchor (Glockner *et al*. 1998), which indicates a putative interaction with membranes. PTPN9 is a phosphatase that localizes on secretory vesicles (Saito *et al*. 2007) and is involved in their fusion control (Huynh *et al*. 2004). Finally, COL4A3BP is a kinase involved in the non-vesicular ER-to-Golgi transport of ceramide (Hanada *et al*. 2003), and may be a phosphorylation target of casein kinase 1-gamma 2 (CSNK1G2; Kumagai *et al*. 2007), which is also present in this submodule (their direct physical interaction tested positive in the Rual *et al*. Y2H screen).

**Figure 4:**
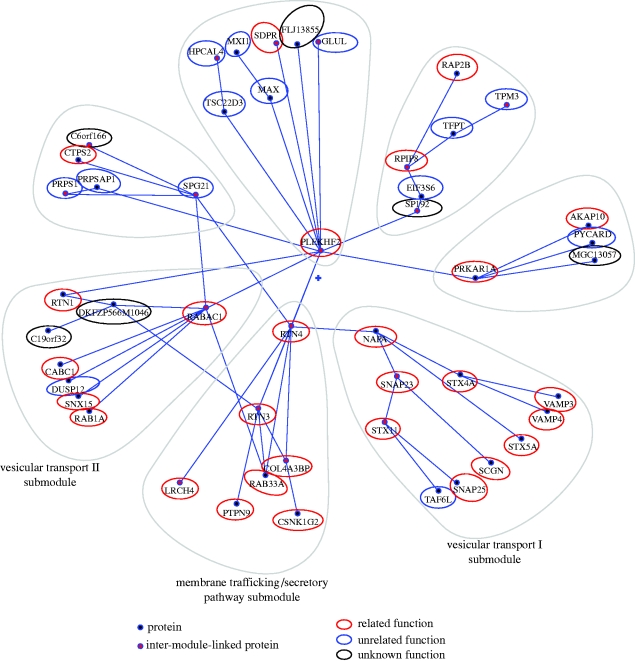
Membrane-interacting proteins module within the human interactome. The local polar map of module 3 in figure 3. Its central theme appears to be membrane-interacting proteins. Note how the submodules (encircled by grey lines) denser in these proteins are found closer together in the circular ordering of the map. See the main text for a biological analysis of this module. To compose this figure, the polar map was exported into an SVG file using Polar Mapper and the graphical highlight around the submodules and relatedness of each protein concerning the module's theme were edited using an external image processing software (Inkscape).

For a different example, we now focus on a submodule we found with Polar Mapper, whose interpretation, although less apparent and necessarily more speculative than in the previous case, may lead to interesting findings. In fact, one of the main interests for pursuing large-scale interactome mapping projects is the hope that they can point researchers in a variety of areas to new leads and directions of study. We designated this submodule (submod. 4) as ‘crosstalk between toll-like receptors (TLRs) and nuclear receptors’. This submodule is found in a module (mod. 2) containing two other submodules also with proteins that fit in this category (figure 5). RARs and RXRs are nuclear retinoid receptors that form RAR/RXR heterodimers in response to retinoids (e.g. retinoic acid), leading to the transcription of specific gene networks (Bastien & Rochette-Egly 2004). RARA, RXRB and RXRG are present in the submodule. SPOP is a poorly characterized protein that is known to bind to and modulate DAXX-mediated transcriptional repression (La *et al*. 2004). SPOP was later identified as an adaptor required for the ubiquitination of DAXX by CUL3-based ubiquitin ligase and consequent degradation by the proteasome (Kwon *et al*. 2006). DAXX is a multifunctional protein that is involved in a wide variety of processes, such as transcription, cell cycle and apoptosis (Salomoni & Khelifi 2006). Unfortunately, DAXX was not present in the Y2H dataset (Rual *et al*. 2005) used in our study. Members of the nuclear receptor superfamily repress proinflammatory programmes of gene expression. The use of specific agonists for nuclear receptors, such as GR (glucocorticoid receptor), LXR (liver X receptors), PPARs (peroxisome proliferator-activated receptors) and, to a lesser extent, RARs, was found to modulate both common and distinct subsets of TLR target genes (Ogawa *et al*. 2005). *DAXX* mRNA expression was over 12-fold upregulated upon stimulation of macrophages using LPS, a well-known TLR agonist. In the presence of specific agonists for GR, LXR α/β and PPARγ, the LPS-induced response was inhibited by 48, 55 and 18%, respectively (Ogawa *et al*. 2005). Unfortunately, this experiment was not done using RARs agonists, since the authors of the study focused on the receptors that modulated the higher number of genes on the initial screening, which does not allow the confirmation of whether RARs are involved in the modulation of *DAXX* expression upon an LPS stimulus. Nevertheless, the link between retinoic acid receptors and DAXX is still present through RXRs, which may also form heterodimeric pairs with other nuclear receptors besides RARs, such as PPARs and LXR (Bastien & Rochette-Egly 2004), which were shown to modulate *DAXX* expression (Ogawa *et al*. 2005). DAXX was also shown to negatively modulate the transcriptional activity of androgen receptor (another nuclear receptor; Lin *et al*. 2004). MYD88 (present in the current submodule) is downstream of several TLRs and is involved in innate immunity signalling (namely through the p38 and JNK pathways) (Ogawa *et al*. 2005) and TLR-induced apoptosis, through an interaction with FADD (Aliprantis *et al*. 2000).

**Figure 5:**
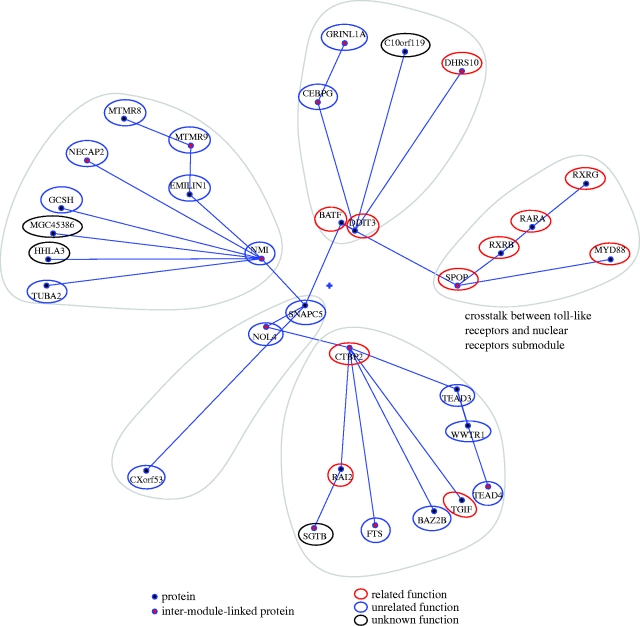
Human interactome module containing the submodule ‘crosstalk between toll-like receptors (TLRs) and nuclear receptors’. The local polar map of module 2 in figure 3. The highlighted submodule contains proteins involved in the crosstalk between toll-like receptors and nuclear receptors. Note that two of the other submodules also contain some proteins in this category. See the main text for a possible functional interpretation of this submodule. This figure was composed as given in the legend to figure 4.

Overall, this preliminary analysis of the CCSB-HI1 dataset served to confirm the presence and relative ease of finding of functional coherent modules in high-throughput interactome data. The latter specific example, found and discussed above, also hints at the likely presence of potentially interesting new leads for a variety of biological research areas in these datasets.

### 4.2. Comparative expression analysis of yeast under hydrogen peroxide and heat shock stress

Microarray-based high-throughput gene expression profiling assays provide, in many regards, a similar challenge to high-throughput interactome mapping assays, namely how to handle the associated large quantities of data and how to extract valuable insights from them. Again, as in the interactome field, the extent to which the level of noise in microarray gene expression assays affects the usefulness of the produced data is a point of dispute. It has been shown already that jointly analysing interaction and expression data can be particularly informative (Jansen *et al*. 2002; de Lichtenberg *et al*. 2005; Palotai *et al*. 2008). Polar Mapper has been set up to allow this in the most straightforward possible fashion: by visually superimposing the expression data (with the standard green/red colour scheme; see Polar Mapper guide in the electronic supplementary material for details on the colouring scheme) over the interactome network. In the following example, we use Polar Mapper, the *S. cerevisiae* filtered yeast interactome (FYI; Han *et al*. 2004) and high-throughput expression data from Gasch *et al*. (2000) to probe for the differences between the yeast oxidative stress (hydrogen peroxide) and heat shock gene expression responses in yeast. The heat shock and oxidative stress responses in yeast are reported to be very similar (Gasch *et al*. 2000). This similarity has been explained as a result of the general environmental stress response (ESR) of yeast (Gasch *et al*. 2000). The main difference between the responses to the two stimuli was identified as being associated with a restricted set of genes related to detoxification processes and reductive reactions in the cell (Gasch *et al*. 2000). We note that the Polar Mapper interactome-based visualization of the expression data can be useful to make apparent other potential differences between these similar expression responses, in particular the differences associated with specific cellular processes, since the modular structure of an interactome appears to reflect such processes.

We start with a Polar Mapper analysis of the observed hydrogen peroxide (HP) stress response by itself. The reader is encouraged to load the associated session file for the yeast gene expression response to hydrogen peroxide,*Yeast_H2O2_fyi.pm* in the electronic supplementary material (showing the expression data superimposed on the interactome network data), and select ‘Island 1’ on Polar Mapper at this stage. The module and submodule names and numeric references in the analysis that follows all refer to that annotated Polar Mapper session. Note that most of the names annotating the modules are lifted from the analysis in Valente & Cusick (2006). Overlaying the mRNA expression data (Gasch *et al*. 2000) obtained from *S. cerevisiae* under HP stress (0.30 mM for 20 min) on the FYI yeast interactome using Polar Mapper, it becomes immediately evident that the genes in several modules of the largest connected component of the interactome behave as ensembles, resulting in entire modules having a clear trend towards repression or induction (figure 6). This pattern is also visible at the submodular level. Some modules present clusters of genes that are up- or downregulated, which are consistent with the submodular grouping (e.g. the cell-cycle control (mod. 23), signalling (mod. 20) and the RNA processing/translation (mod. 18) modules). A very robust repression of ribosomal transcripts (see the large 60S (mod. 6) and small 40S (mod. 25) ribosomal subunit modules) is apparent. This type of response to stress (including oxidative stress) is well known (Warner 1999; Gasch *et al*. 2000; Marques *et al*. 2006). Modules composed of genes involved in mRNA-related processes are repressed as well, which is evident in the exosome (mod. 8) and the spliceosome (mod. 17) modules. Additionally, translation has also been reported to be repressed during stress (Gasch *et al*. 2000; Shenton *et al*. 2006), which can be readily identified in the translation initiation complex module (mod. 21) and the translation/translation initiation submodule (mod. 18, submod. 74). Genes involved in mitosis are also repressed, as seen in the anaphase-promoting complex (APC) submodule (mod. 14, submod. 55), the chromosome condensation/segregation module (mod. 13), the cytokinesis/chromosome segregation module (mod. 22) and submodule 99 of the cell-cycle control module (mod. 23; analysed later in greater detail). These results are consistent with the reports that HP induces a G2/M arrest in yeast (Shapira *et al*. 2004). Conversely, some modules are clearly upregulated. There is induction of genes involved in protein degradation, namely proteasomal genes (see the proteasomal regulatory complex and the proteasomal catalytic complex modules, modules 4 and 3, respectively), which is in agreement with other studies using HP as a stressor on yeast (Marques *et al*. 2006). The DNA repair submodule (mod. 14, submod. 57) is also upregulated. It is known that HP and other oxidative stress inducers may generate DNA damage and induce the cellular DNA repair mechanisms (Gasch *et al*. 2000; Ikner & Shiozaki 2005). Other modules present upregulated submodules, but, overall, the notion that gene repression is predominant under oxidative stress (Gasch *et al*. 2000) becomes rather evident upon visualization of the overall expression profile superimposed on the interactome (figure 6). The cell-cycle control module (mod. 23) is an interesting example of one module that does not show a clear trend towards up- or downregulation. However, by zooming in at the module level, it becomes evident that it contains submodules that are induced, while others are repressed. One of the submodules, named the G1/S submodule (submod. 98), contains mainly proteins that are involved in the G1- and S-phases of the cell cycle. This submodule is upregulated, which is in agreement with the results showing that yeast cells are able to progress through G1/S upon HP challenge (Shapira *et al*. 2004). Interestingly, this study also showed that the S-phase duration was slightly prolonged compared with untreated cells. This fact is consistent with the observation that, despite many G1/S transcripts being upregulated, *CDC6* is downregulated. This gene is essential for DNA replication initiation during the S-phase (Speck *et al*. 2005). Several G2/M-related proteins can be found in submodule 99 in which, with the exception of *CAK1*, all the G2/M-related genes are downregulated. This reinforces the strong effect of HP on this phase of the cell cycle.

**Figure 6:**
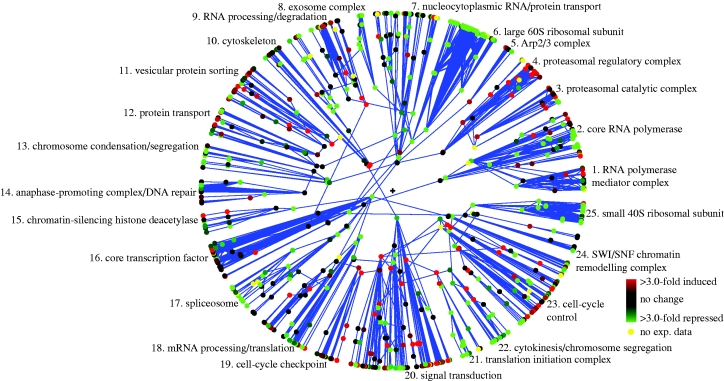
Hydrogen peroxide oxidative stress gene expression response of *S. cerevisiae*. The polar map showing *S. cerevisiae* hydrogen peroxide oxidative stress mRNA expression data (0.30 mM for 20 min) from Gasch *et al*. (2000) overlayed on the largest connected component of the yeast protein interaction network, based on the FYI dataset (741 proteins and 1752 interactions; Han *et al*. 2004). The colours show mRNA fold induction relative to control unchallenged cells. See the main text for a description of this oxidative stress response.

We now compare the heat shock (HS; 25–37°C for 20 min, data from Gasch *et al*. 2000) and HP stress responses of yeast. A key technique we use here is to visualize the HP stress expression response *relative* to the heat shock (HS) stress expression response. This is done by loading into Polar Mapper the difference between the log expression data for the HP and HS responses (figure 7; Polar Mapper session *Yeast_H2O2-Heat_Shock_fyi.pm, ‘Island 1*’ in the electronic supplementary material). For the most part, the trend towards induction or repression in any given interactome module is the same under both HP and HS stresses. However, by looking at the above difference, the modules that are *more intensely* induced or *more intensely* repressed under HP than under HS become evident, since they retain their net original colour associated with up- (red) or downregulation (green), respectively, observed in the HP-only image (figure 6). Conversely, when the colour trend is reversed between figures 6 and 7, it must be that the induction/repression is more intense in HS than in HP. It becomes therefore simple to visually identify the modules and submodules that may be more affected or regulated by each specific stress.

**Figure 7:**
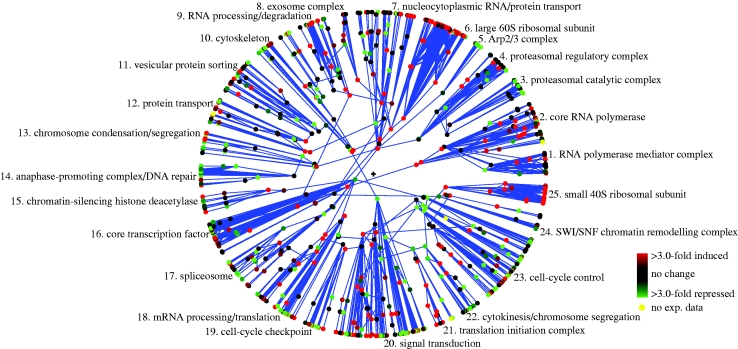
Hydrogen peroxide oxidative stress versus heat shock gene expression response in *S. cerevisiae*. Analogous to figure 6, except that the colours now show hydrogen peroxide oxidative stress mRNA fold induction *relative* to heat shock stress. See the main text for analysis.

From figures 6 and 7, it becomes evident that some modules are more strongly up- or downregulated by each particular stimulus (table 1, HP versus HS). During the HP challenge, the repression of the G2- and mitosis-related modules is stronger than in HS stress (see the chromosome condensation/segregation module (mod. 13), the APC submodule (submod. 55) and the cytokinesis/chromosome segregation module (mod. 22)). The cell-cycle control module (mod. 23) provides us with further information: the G2/M inducers are more repressed or less upregulated in HP than in HS (table 2). This suggests that HP has an effect on the cell cycle, namely on the G2/M phase of the cell cycle. This is in agreement with published data, reporting the existence of a G2/M block after HP stress (Shapira *et al*. 2004), but not during HS (Li & Cai 1999). In fact, this heat shock study (Li & Cai 1999) reports that HS induces a transient G1/S arrest. This is supported by the data showing that several key G1/S genes are differentially expressed between HP and HS: the expression of the G1/S inhibitor *SIC1* remains unchanged in HP and is upregulated in HS; conversely, the G1/S inducers *CLB5* and *CLB6* (critical for DNA replication) remain unchanged in HP and repressed in HS; *SWI4* (involved in G1/S progression) is also less strongly upregulated in HS than in HP. Although there are some exceptions and also some incomplete data, in general, the G1/S gene expression comparison on this module fits the experimental data (table 2). Interestingly, the DNA repair submodule (submod. 57; table 1) is more strongly induced in HP than in HS, which is also in line with the general notion that direct DNA damage is extremely important during oxidative stress (Wang *et al*. 1998; Ikner & Shiozaki 2005; Pan *et al*. 2006). Overall, this analysis suggests that there is a tighter control on proteins and RNA synthesis and degradation, as well as on G1/S progression upon heat shock; whereas mitotic control and DNA repair are more strongly regulated during HP stress (table 1).

**Table 1:** Summary of the main modules and submodules identified using Polar Mapper as being more strongly upregulated or downregulated under the hydrogen peroxide or heat shock stimulus in yeast. (See the main text for analysis.)

	hydrogen peroxide	heat shock
stronger downregulation	chromosome condensation/segregation	large 60S ribosomal subunit
	cytokinesis/chromosome segregation	small 40S ribosomal subunit
	anaphase-promoting complex (submod. 55)^a^	translation initiation complex
		exosome complex
		nucleoplasmic RNA/protein transport
		core RNA polymerase
stronger upregulation	DNA repair submodule (submod. 57)^b^	protein folding submodule (submod. 100)
	G1/S submodule (submod. 98)	proteasomal catalytic complex

a The APC submodule does not show a clear trend towards downregulation during heat shock.

b The DNA repair submodule, in fact, shows a downregulation trend during heat shock.

**Table 2:** Comparative expression of cell-cycle genes present in the cell-cycle control module upon hydrogen peroxide treatment and heat shock in yeast. (See the main text for analysis.)

	HP	HS
*G1/S inhibitors*		
SIC1	unchanged	+
*G1/S inducers*		
CLB5	unchanged	−
CLB6	unchanged	−
SWI4^a^	++	+
SWI6^b^	−−	−
MBP1	−	n.a.
CLN1	−	−−
CLN2^b^	−−	−
*G2/M inducers*		
CKS1	n.a.	−
CLB1	−−	−
CLB2	−	n.a.
CLB3	−−	−
CLB4	−	−
CAK1	+	++

a SWI4 is involved in DNA synthesis and also in DNA repair.

b Genes whose expression is opposite to the trend of most genes in the submodule. CDC28 is also present in this submodule, but it is involved in both G1/S and G2/M progressions.

Owing to the small number of chaperones present in the FYI dataset that we analysed, a relevant matter we did not explore is the collective change induced by stress in chaperones and their low affinity, but fundamental, transient interactions (Korcsmáros *et al*. 2007). For instance, similarly, based on a combined gene expression and protein interaction data analysis, it has been hypothesized that, in yeast, cellular stress leads chaperones to become more central in the interactome (Palotai *et al*. 2008). A next step in validating and refining some of the ideas related to the dynamic nature of the interactome will probably require the actual experimental testing of protein–protein interactions in cells under different conditions. It has also recently been shown that, in eukaryotic cells, in contrast to bacteria, there are two distinct chaperone networks, one being involved in de novo protein folding (coupled to translation) and the other in the rescue of stress-denatured proteins (Albanèse *et al*. 2006). In this regard, from the 14 chaperones identified as translation coupled by Albanèse *et al*. (2006), seven out of the eight present in the FYI dataset that we analysed belonged to the same interactome submodule (mod. 23, submod. 100; the exception being one protein present in mod. 23, submod. 101). Conversely, out of the 20 chaperones categorized as stress coupled by Albanèse *et al*. (2006), the four present in the FYI dataset were all grouped together by Polar Mapper in a distinct module from the translation-coupled one (mod. 10, submod. 37). The placement in the interactome of these few chaperones present in the FYI dataset seems therefore to be consistent with the two separate functional chaperone classes identified in the study of Albanèse *et al*. (2006).

Overall, our case study showed how this graphical platform, combining expression and interaction data, can aid in a first-pass analysis and organization of expression data. Note, in particular, how it allowed a faster identification of relevant cellular processes, giving hints on biological processes that should be subjected to a more detailed study. It also serves to note that, in spite of the respective assay reliability limitations, the present-day high-throughput interaction and expression data can already be valuable resources in biological research.

## 5. Summary

This paper introduces Polar Mapper, a computational application centred around the polar map visualization of protein interaction networks. It is meant to be a practical, ready-to-use auxiliary tool in biological research work that involves the analysis of protein interaction networks. A second objective of this paper is to make available to the scientific community a reusable implementation of the polar map algorithm in order to contribute to the ongoing development of improved biological network visualization tools. To this end, the source code and documentation are open and freely available to the community. Finally, although visualization of protein interaction networks was the primary motivation for this application, we note that it may probably be profitably employed in the display of many other kinds of binary interaction data networks.

## 6. Availability

Polar Mapper is implemented in Java and may be used within several platforms and environments, as long as a Java virtual machine installation is provided. Binaries and source code, as well as documentation, are available both as the electronic supplementary material at the *Journal of the Royal Society Interface* website and at the Polar Mapper website (http://kdbio.inesc-id.pt/software/polarmapper).
